# Comparative network analysis via differential graphlet communities

**DOI:** 10.1002/pmic.201400233

**Published:** 2014-12-15

**Authors:** Serene W H Wong, Nick Cercone, Igor Jurisica

**Affiliations:** 1 Department of Computer Science and Engineering, York UniversityToronto, Canada; 2 Princess Margaret Cancer Centre, TECHNA Institute for the Advancement of Technology for Health, UHNToronto, Canada; 3 Departments of Computer Science and Medical Biophysics, University of TorontoToronto, Canada

**Keywords:** Comparative network analysis, Differential graphlet communities, Non-small cell lung cancer, Systems Biology

## Abstract

While current protein interaction data provides a rich resource for molecular biology, it mostly lacks condition-specific details. Abundance of mRNA data for most diseases provides potential to model condition-specific transcriptional changes. Transcriptional data enables modeling disease mechanisms, and in turn provide potential treatments. While approaches to compare networks constructed from healthy and disease samples have been developed, they do not provide the complete comparison, evaluations are performed on very small networks, or no systematic network analyses are performed on differential network structures. We propose a novel method for efficiently exploiting network structure information in the comparison between any graphs, and validate results in non-small cell lung cancer. We introduce the notion of differential graphlet community to detect deregulated subgraphs between any graphs such that the network structure information is exploited. The differential graphlet community approach systematically captures network structure differences between any graphs. Instead of using connectivity of each protein or each edge, we used shortest path distributions on differential graphlet communities in order to exploit network structure information on identified deregulated subgraphs. We validated the method by analyzing three non-small cell lung cancer datasets and validated results on four independent datasets. We observed that the shortest path lengths are significantly longer for normal graphs than for tumor graphs between genes that are in differential graphlet communities, suggesting that tumor cells create "shortcuts" between biological processes that may not be present in normal conditions.

## 1 Introduction

Most cancers lack effective early disease markers, prognostic, and predictive signatures, primarily due to tumor heterogeneity. As a result, we fail treating cancer heterogeneity due to multiple ways cancer initiates and develops treatment resistance. Models that represent these differences and the underlying molecular mechanism in cancer enhance the possibility in characterizing and in turn treating cancer successfully.

Current protein–protein interaction (PPI) information is a rich resource for molecular biology research, but it lacks the condition-specific context for PPIs. In order to understand diseases, gene expression profiling can be used. Differential expression studies that compare gene expression levels between healthy and affected tissues have been developed [1]. Differential expression studies usually involve detecting statistical significance changes to the mean expressions of individual genes [2]. Some studies associated changes in mean expression levels in gene groups or pathways with disease phenotypes [1]. However, useful prognostic signatures are not necessarily the most differentially expressed genes [3]. Differential coexpression approaches that compare coexpression patterns between healthy and diseased samples have been developed. Studies have identified several highly differentially coexpressed transcriptional regulators involved in cancer, but their mean expressions did not change much [1].

Identification of differences between healthy and diseased tissues is important, but the difference should not be limited to gene groups. Difference in network structure is essential as studies have shown that systematically analyzing structural properties of biological networks can bring forth important insights, for example, determining the relationship between network topology and protein functions, or network topology and the underlying disease mechanism (e.g. [4–6]). These results have to be interpreted carefully as trends can be due to literature bias; however, they suggest that there is a relationship between structures and functions in networks that needs to be explored further.

Importantly, network-based approaches have been successful in identifying subnetworks for classification, for recovering of known and uncovering of novel biological functions. For example, Ideker et al. showed that top-scoring subnetworks overlap well with known regulatory mechanisms [7]. Chuang et al. showed that identified subgraphs were more reproducible, and better predict breast cancer metastasis than individual genes [8]. Subnetworks have also been shown to be effective biomarkers in the prediction of aging [9]. Thus, identification of differences between healthy and diseased tissues should include differences in network structures.

Several approaches to compare coexpression networks constructed from healthy and disease samples have been developed, e.g. [10–12]. Other approaches use dependency networks to compare healthy and disease networks, e.g. [13,14]. The most straightforward way for such network comparison is to use the connectivity of each gene in the healthy and disease network [1]. Previous methods used diverse approaches to compare two networks: (1) simple gene connectivity or its variations; (2) edge or the mean edge weight between groups. Although network comparison provides important information about disease mechanism, it has not yet been used to its full potential. Importantly, differential network structures need to be systematically analyzed and characterized. We propose a novel method that uses network structure information to compare any graphs.

In order to compare and characterize different complex networks, we can use global or local network properties. Global network properties examine the overall network, while local network properties focus on local structures or patterns of the network [15]. Commonly used global network properties include degree distribution, diameter and clustering coefficient; however, these measures do not sufficiently capture the structural characteristics of biological networks [16]. Thus, more sensitive local structure measurements have emerged. *Graphlets* are all nonisomorphic connected induced graphs on a specific number of vertices [17]. By definition, they have the ability to capture all the local structures on a certain number of vertices.

Relative graphlet frequency distance [18] and graphlet degree distribution agreement [15] have been developed as local network structure measures. Both measures return a scalar for the difference between two graphs. Existing graphlet-based measures are useful for comparing graphs efficiently, since only scalars need to be evaluated. However, our aim is to make the most of graphlet information, and use it to further characterize network structure differences between any graphs. We propose a novel method that not only lists graphlets in graphs *A* and *B*, but identifies and annotates deregulated subgraphs that differ between the two graphs. Furthermore, our approach circumvents the exponential growth of computation required as the graphlet size increases, and enables systematic characterization of protein communities with larger size, which provide stronger biological context. Previous graphlet-based approaches considered two to five node graphlets, but the size of our detected deregulated communities can be much larger than the size of individual graphlets.

We introduce the notion of differential graphlet community to detect deregulated subgraphs between any graphs such that the network structure information is exploited. The differential graphlet community approach overcomes a limitation of existing approaches (e.g. [11,12]), importantly, it has the ability to include a gene into more than one deregulated subgraph. The ability for overlapping differential graphlet communities is important because genes can have multiple functions under different biological contexts. While the differential graphlet community approach is generic, we evaluated it on three non-small cell lung cancer (NSCLC) datasets. Our results show that the difference in network topology between normal and tumor graphs provides insights to the underlying molecular mechanism in NSCLC. In particular, a trend that the shortest path lengths are longer for normal graphs than for tumor graphs in differential graphlet communities is observed, suggesting that tumor cells can create shortcuts between biological processes that may not be present in normal conditions. Examples of shortcuts that are observed, and are in agreement with known mechanism in literature include the cross-talk between the Jak-STAT and *NF-kappaB* pathways or *STAT3* signaling enabling cross-talk among tumor and immune cells, resulting in an immunosuppressive network.

## 2 Materials and methods

### 2.1 Graphlet approach

**Figure 1:**
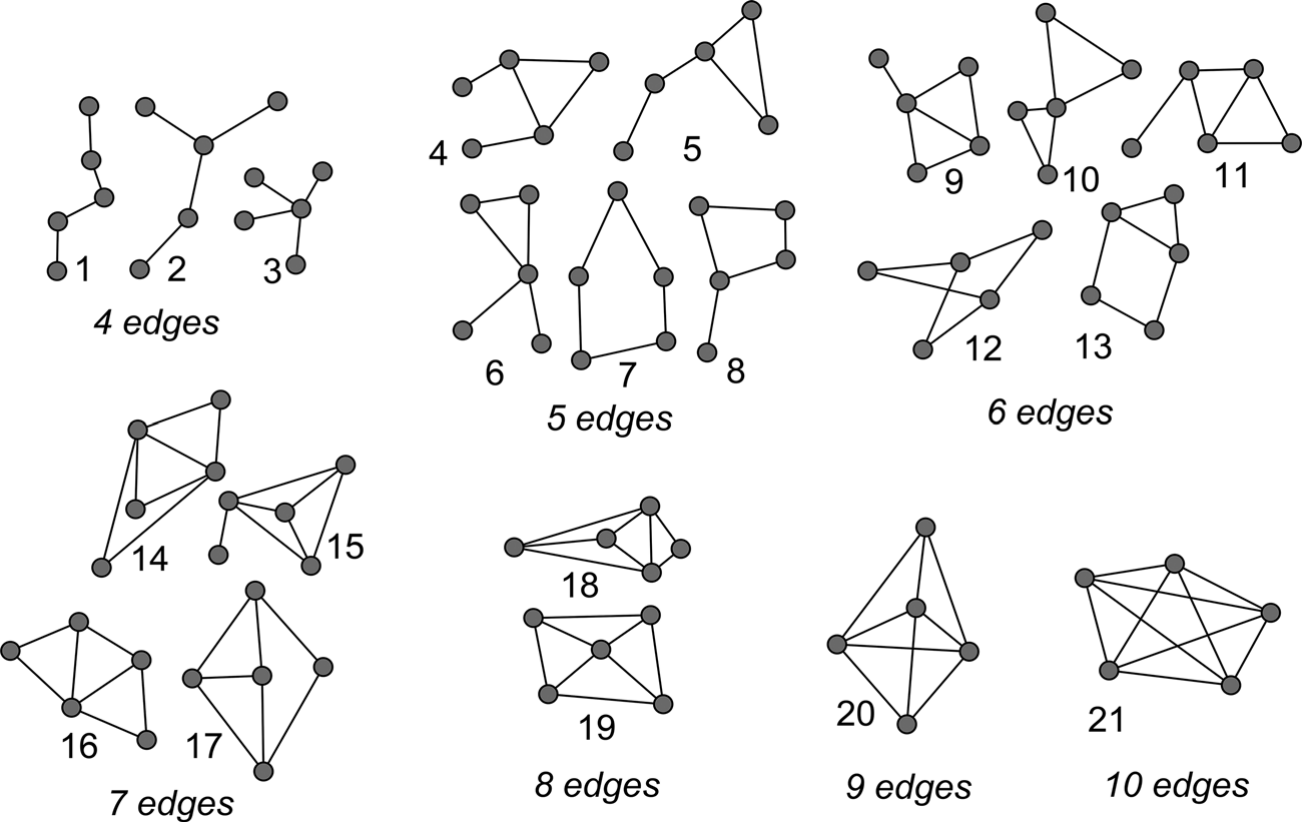
All twenty-one 5-node graphlets, all nonisomorphic, connected, induced graphs on five vertices.

We have proposed a graphlet approach to systematically extract network structure differences between normal and NSCLC graphs [19]. We enumerate all *n*-node graphlets in normal graphs and NSCLC graphs. This involves the subgraph isomorphism problem, which is NP-complete [20]. As *n* increases, the number of different types of subgraphs increases exponentially [18], and the time and memory needed to determine isomorphic subgraphs increases exponentially as well [21]. The use of differential graphlet communities can help circumvent this exponential growth of computation and space required. Importantly, the number of genes that function together is often more than a few. Previous approaches considered 2*−*5 node graphlets [15,18]. Since exploring protein communities with larger size provides stronger biological context, the largest feasible graphlet size with respect to previous graphlet-based measures is chosen; that is, *n* is 5. Figure 1 shows all 5-node graphlets. The graphlet approach is systematic because all 5-node graphlets from the normal and NSCLC graphs are enumerated, and no subgraph of size 5 will be missed.

The graphlet approach provides us with the protein wiring information that differentiates between normal and NSCLC graphs, and thus may provide insights to the underlying mechanisms and eventually lead to novel lung cancer treatments.

### 2.2 Differential graphlet community

Enumerating 5-node graphlets ensures that all nonisomorphic connected induced graphs on five nodes will be considered. However, the number of genes that function together is often more than 5. Furthermore, any two graphlets, *A* and *B* can potentially have four nodes that overlap. Thus, we extend the approach to consider graphlet communities with a goal to identify the difference in the properties of networks between different graphs—in this paper, between normal and tumor graphs.

Palla et al. [22] defines a community as the union of all *k*-cliques such that one can reach to another by a chain of adjacent *k*-cliques. A *k*-clique is a complete graph with *k* vertices. Adjacent *k*-cliques are *k*-cliques that share *k −* 1 nodes. A differential graphlet community is defined as the union of all *k*-graphlets such that one can reach to another by a chain of adjacent *k*-graphlets. Adjacent *k*-graphlets are graphlets that share *k −* 1 nodes. Since all 5-node graphlets are enumerated, *k* is 5 for the purpose of this paper.

The differential graphlet community approach detects deregulated subgraphs that differ between two graphs. There are several advantages to the differential graphlet community approach. First, the proposed approach has the ability to include a gene into more than one deregulated subgraph. The ability for overlapping differential graphlet communities is important because genes can have multiple functions in biological systems. Second, the differential graphlet community approach circumvents the exponential growth of computation required as the graphlet size increases, and enables the systematically exploring of protein communities with larger size that provide stronger biological context. Thus, although the size of each graphlet is 5, the sizes of differential graphlet communities can be much larger. Third, no predetermined size or number of deregulated subgraphs are required as input to the method, size, and the number of communities are determined automatically.

We describe the differential graphlet approach in this section. Further information on the construction of coexpression graphs, graph theoretical terms, and the implementation are in Supporting Information.

#### 2.2.1 Construction of coexpression graphs

While the approach is generic, we evaluated it on three NSCLC gene expression datasets. Two coexpression graphs for each dataset, a normal, and a tumor graph, are generated using normal and tumor samples, respectively (details are provided in Supporting Information).

#### 2.2.2 Enumeration of graphlets

For each dataset, given a normal and a tumor graph, all 5-node graphlets are enumerated. We separate the enumeration of 5-node graphlets into three categories:

(1) NORMAL: graphlets that are only in the normal graph.
(2) BOTH: graphlets that are in the normal and tumor graphs, but with structural differences.
(3) TUMOR: graphlets that are only in the tumor graph.

We focus on graphlets that are in the tumor category, and those that have the same membership across all three datasets. Differential graphlet communities are then computed for the extracted graphlets. The differential graphlet community analysis identifies interactions between proteins that are deregulated in tumors. Deregulations are seen from the difference in network structures between the normal and tumor graph.

### 2.3 Datasets

We applied our approach to three NSCLC datasets [23–25], referred to as Hou, Su, and Landi in this paper. Datasets have been selected based on the number of normal and tumor samples they contain, and were downloaded from Gene Expression Omnibus database [26].

We used four independent NSCLC gene expression datasets [27–30] to validate our results (referred to as Lu, Sanchez, Okayama, and Girard, respectively).

Supporting Information Tables 1 and 2 provide additional details on the seven datasets.

### 2.4 Notations

Let *Hou_N_, Su_N_, Landi_N_* denote the normal graphs for Hou, Su, and Landi, respectively. Similarly, let *Hou_T_, Su_T_, Landi_T_* denote the tumor graphs for Hou, Su, and Landi, respectively.

Let *g*_*T*−*Hou*_, *g*_*T*−*Su*_, *g*_*T*−*Landi*_ denote the set of graphlets that are in the tumor category for datasets Hou, Su, and Landi, respectively. Let *M*_*TALL*_ denote the set containing sets of five vertices such that *V*(*h*) = *V*(*s*) = *V*(*l*) for some *h* ∈ *g*_*T*−Hou_, *s* ∈ *g*_*T*−*Su*_, *l* ∈ *g*_*T*−*Landi*_. *|M*_*TALL*_*|* is the number of graphlets that have the same membership across all three datasets in the tumor category.

Differential graphlet communities are then computed on *g*_*T*−*Hou*_ for all *h* ∈ *g*_*T*−*Hou*_, *g*_*T*−*Su*_ for all *s* ∈ *g*_*T*−*Su*_, *g*_*T*−*Landi*_ for all *l* ∈ *g*_*T*−*Landi*_ such that *V*(*h*), *V*(*s*), *V*(*l*) ∈ *M*_*TALL*_.

We have identified three differential graphlet communities for each dataset, referred to as: *dGC*_*Hou*_*i*, *i* ∈ {1, 2, 3} for Hou, *dGC*_*Su*_*i, i* ∈ {1, 2, 3} for Su and *dGC*_*Landi*_i, *i* ∈ {1, 2, 3} for Landi. Importantly, note that *V*(*dGC*_*Hou*_*i*) = *V*(*dGC*_*Su*_*i*) = *V*(*dGC*_*Landi*_*i*), *i* ∈ {1, 2, 3}, respectively, and thus the computation returns the same number of differential graphlet communities for each dataset.

All shortest paths are computed between all vertex pairs in *V*(*dGC*_*Hou*_*i*), *i* ∈ {1, 2, 3} for *Hou*_*N*_ and for *Hou_T_*. All shortest paths are computed between all vertex pairs in *V*(*dGC*_*Su*_*i*), *i* ∈ {1, 2, 3} for *Su_N_* and for *Su_T_*. Finally, all shortest paths are computed between all vertex pairs in *V*(*dGC*_*Landi*_ *i*), *i* ∈ {1, 2, 3} for *Landi_N_* and for *Landi_T_*.

Let *dGCsp*_*HouN*_*i, i* ∈ {1, 2, 3} denote the shortest path graph for differential graphlet community *i* for dataset Hou in Hou's normal graph. *dGCsp*_*HouN*_*i, i* ∈ {1, 2, 3} contains all shortest paths in *Hou*_*N*_ between all vertex pairs in *V*(*dGC*_*Hou*_*i*), *i* ∈ {1, 2, 3}. Let *dGCsp*_*HouT*_*i*, *i* ∈ {1, 2, 3} denote the shortest path graph for differential graphlet community *i* for dataset Hou in Hou's tumor graph.

### 2.5 Shortest path distribution

After obtaining deregulated subgraphs, comparing network structures is important for the understanding of disease mechanisms. In order to better utilize network structure information obtained from the deregulated subgraphs, we computed shortest path distributions on differential graphlet communities.

Visualization of differential graphlet communities in Network Analysis, Visualization and GrAphing, TORonto [31] shows that there are fewer vertex pairs *xy* such that *x* is adjacent to *y* among vertices in *V*(*dGC*_*Hou*_*i*), *i* ∈ {1, 2, 3} for *Hou*_*N*_ than in *dGC*_*Hou*_*i, i* ∈ {1, 2, 3}, respectively. Similar results are observed for Su and Landi datasets. To quantify these observations, we performed a systematic shortest path distribution analysis. All shortest paths are computed in the normal and tumor graphs for all vertex pairs in differential graphlet communities.

Shortest path distributions are computed for:

(1) *dGCsp*_*HouN*_*i*, *i* ∈ {1, 2, 3} and *dGCsp*_*HouT*_*i*, *i* ∈ {1, 2, 3};
(2) *dGCsp*_*SuN*_*i*, *i* ∈ {1, 2, 3} and *dGCsp*_*SuT*_*i*, *i* ∈ {1, 2, 3};
(3) *dGCsp*_*LandiN*_*i*, *i* ∈ {1, 2, 3} and *dGCsp*_*LandiT*_*i*, *i* ∈ {1, 2, 3}.

Significance of shortest path distribution differences between normal and tumor graphs is determined by the Mann–Whitney test. A constant *C* is used to replace infinity distance (i.e. nonreachable vertices). By the nature of the Mann–Whitney test, results from different *C*s will be the same if *C* is greater than all noninfinity lengths in the compared shortest path distributions. Thus, without loss of generality, *C* is set to be 100 as the maximum shortest path length is 12.

### 2.6 Pathway and GO analysis

In order to gain biological insights from network structures of the differential graphlet communities, and to test whether edges in differential graphlet communities are within a pathway or across pathways, nodes were overlapped with pathways and GO. Pathway databases used include Encyclopedia of *Homo Sapiens* Genes and Metabolism [32], Kyoto Encyclopedia of Genes and Genomes (KEGG) [33], National Cancer Institute-Pathway Interaction Database [34], Reactome [35], and The Cancer Cell Map [36]. KEGG was downloaded on Feb 2011; remaining databases were downloaded from Pathway Commons [37] on Aug, 2012. Annotations for GO ontology—biological process were downloaded from Quick GO from European Bioinformatics Institute [38] on August, 2012.

The intersection of *dGCsp*_*HouT*_*i*, *dGCsp*_*SuT*_*i*, and *dGCsp*_*LandiT*_*i* is taken for *i* ∈ {1, 2, 3}, and is denoted as *dGCsp*_*ALL*_*i, i* ∈ {1, 2, 3}. *V(dGCsp*_*ALL*_*i), i* ∈ {1, 2, 3} were intersected with individual pathways and GO biological processes.

## 3 Results and discussion

**Figure 2:**
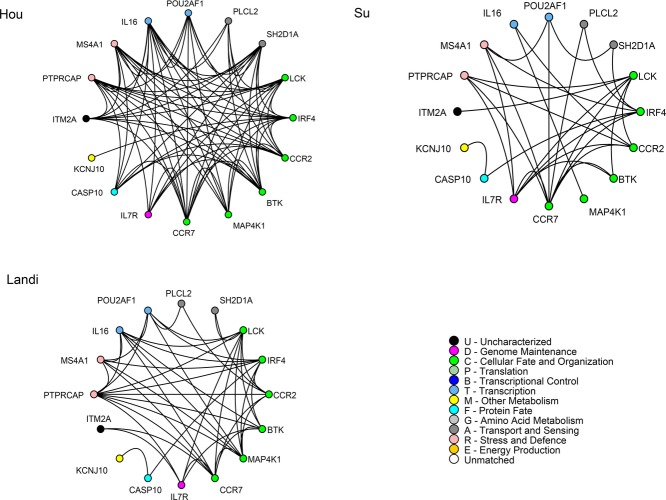
*dGC*_*Hou*_1, *dGC*_*Su*_1, and *dGC*_*Landi*_1 are shown. Edges connect coexpressed genes. Nodes are sorted and colored based on GO biological function.

We identified three differential graphlet communities for each dataset; for all three differential graphlet communities, for all seven datasets, we observed a trend that the shortest path lengths are shorter for tumor graphs compared to normal graphs. All nodes and edges of differential graphlet communities *dGC*_*Hou*_*i*, *dGC*_*Su*_*i*, and *dGC*_*Landi*_*i, i* ∈ {1, 2, 3} are presented in Fig. 2 and Supporting Information Figs. 1 and 2. Note that the difference in wiring in individual datasets could be due to the difference in disease stage as well as the difference in histology.

We also present the comparisons of shortest path distributions for:

(1) *dGCsp*_*HouN*_*i* versus *dGCsp*_*HouT*_*i* for *i* ∈ {1, 2, 3};
(2) *dGCsp*_*SuN*_*i* versus *dGCsp*_*SuT*_*i* for *i* ∈ {1, 2, 3};
(3) *dGCsp*_*LandiN*_*i* versus *dGCsp*_*LandiT*_*i* for *i* ∈ {1, 2, 3}.

For readability, simpler terms are used in the Figures. For example, shortest path distribution for Landi for *dGC*1 refers to the comparison of the shortest path distributions between *dGCsp*_*LandiN*_*1* and *dGCsp*_*LandiT*_*1*.

**Figure 3:**
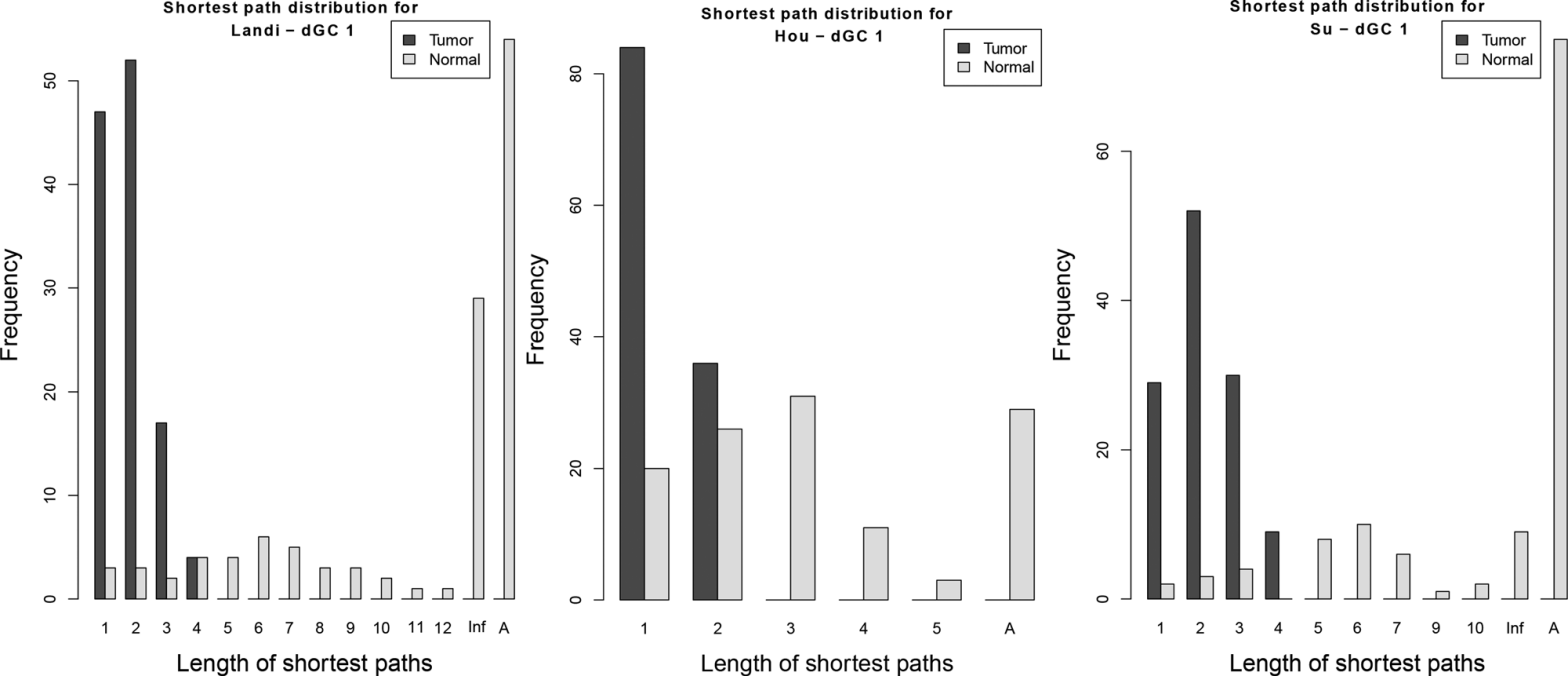
Shortest path distributions for *dGC*1 for Landi, Hou, and Su datasets. Inf represents shortest path between unreachable nodes. A is the number of node pairs that have infinity as the distance due to the absence of nodes in the graph.

**Figure 4:**
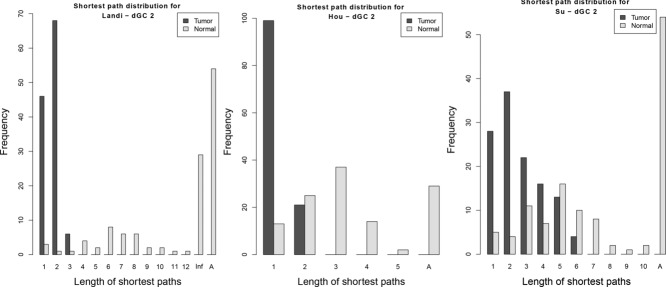
Shortest path distributions for *dGC*2 for Landi, Hou, and Su datasets. Inf represents shortest path between unreachable nodes. A is the number of node pairs that have infinity as the distance due to the absence of nodes in the graph.

Figures 3, 4 and Supporting Information Fig. 3 show that for all three datasets, for all three differential graphlet communities, tumor graphs have shorter shortest paths than normal graphs; the median of shortest path lengths in normal is significantly larger compared to tumor graphs (adjusted *p* values *≤* 1.13E *–* 20; one-sided Mann–Whitney test). This suggests that tumor cells may cause cross-talk between biological processes that usually does not exist under normal conditions.

To further validate the observed trend, we used four independent NSCLC datasets—Lu, Sanchez, Okayama, and Girard [27–30]. In all four datasets, for all three differential graphlet communities, the observed trend is confirmed: tumor graphs have shorter shortest paths compared to normal graphs; the median of shortest path lengths in normal is significantly larger than tumor graphs (adjusted *p* values ≤ 2.61E *–* 13; one-sided Mann–Whitney test). Supporting Information Figs. 4, 5, and 6 show the observed trend for different datasets for differential graphlet community 1, 2, and 3, respectively.

Thus, for all seven datasets, for all three differential graphlet communities, we observed a trend that the shortest path lengths are shorter for tumor graphs compared to normal graphs; the median of shortest path lengths in normal is larger than that of tumor graphs, as determined using the one-sided Mann–Whitney test (adjusted *p* values *≤* 2.61E *–* 13).

### 3.1 Biological meaning of differential graphlet communities

From the shortest path distributions across all seven datasets and all three differential graphlet communities, we observed a trend that the shortest path lengths are longer for normal graphs than for tumor graphs. The observed trend suggests that tumor cells create shortcuts between biological processes that are usually not connected under normal conditions.

In order to test whether edges in differential graphlet communities are within a pathway or across pathways, nodes in differential graphlet communities were overlapped with pathways and GO biological processes, and are presented in Supporting Information Tables *M* 1*−*9 in Supporting Information Additional file 2.

#### 3.1.1 A proof-of-concept

**Figure 5:**
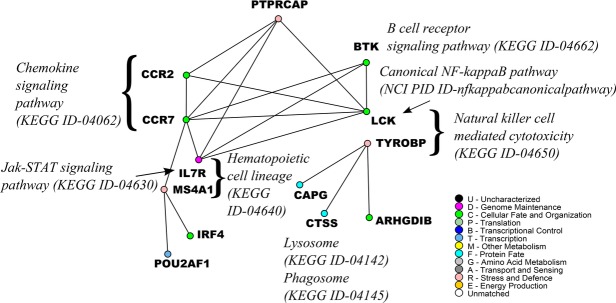
An example from *dGCsp*_*ALL*_2. Edges link coexpressed genes. Nodes are colored based on GO biological function. *IL7R* belongs to the Jak-STAT signaling pathway and the hematopoietic cell lineage. *LCK* belongs to the canonical *NF-kappaB* pathway and the natural killer cell mediated cytotoxicity.

We use an example from *dGCsp*_*ALL*_2 as a proof-of-concept to demonstrate that the differential graphlet community approach provides insights into the underlying mechanism, and potential novel treatments for NSCLC. Figure 5 presents *dGCsp*_*ALL*_2 labeled with pathway information, and it shows that many edges in *dGCsp*_*ALL*_2 are across different pathways suggesting cross-talk between them.

In *dGCsp*_*ALL*_2, there are many edges crossing between members of the chemokine signaling pathway, Jak-STAT signaling pathway, Canonical *NF-kappaB* pathway, and the B-cell receptor signaling pathway. It has been reported that Jak-STAT signaling pathway and Canonical *NF-kappaB* pathway have *STAT3* and *NF-kappaB* “collaborating” in cancer [39]. The activation of *STAT3* and *NF-kappaB* as well as the interaction between them are important for controlling the communication between a malignant cell and its microenvironment. Often, *STAT3* and *NF-kappaB* are basally active in neoplastic cells. A global profiling of mouse lung cells showed that *STAT3* controlled the expression of a large number of genes, and some *NF-kappaB* target genes were among them [40]. Genes that are controlled by *STAT3* and *NF-kappaB* include chemokines, *PAI-1*, *Bcl3*, *Bcl2*, *GADD45β*, and *SOCS3*. This suggests that *STAT3* and *NF-kappaB* pathways work together for the induction of specific groups of genes [39].

*CCR2* and *CCR7* are chemokine receptors in the chemokine signaling pathway identified in *dGCsp*_*ALL*_2. Genes that encode chemokines are among targets for *STAT3* and *NF-kappaB* [39]. Chemoattractants are crucial for recruiting and renewing various cells in the tumor microenvironment. In particular, *CCL2*, a *CCR2* ligand, controls the enrollment of myeloid cells, which induce tumor-associated macrophage or myeloid-derived suppressor cells (MDSC) [39]. In the tumor microenvironment, tumor-associated macrophage can promote tumor and MDSC can suppress T cells [41]. Another chemokine receptor in *dGCsp*_*ALL*_2 is *CCR7*. *CCL19* /*CCL21* /*CCR7* play a role in attracting immunosuppressive T-regulatory cells [42]. Therefore, *STAT3* and *NF-kappaB*, through the regulation of chemokine synthesis, can determine which groups of immune cells are active in the tumor microenvironment.

Not only is *STAT3* observed to have cross-talk with *NF-kappaB*, *STAT3* signaling also enables cross-talk among tumor and immune cells, resulting in an immunosuppressive network [43]. This cross-talk via *STAT3* signaling involves hematopoietic progenitor cells, and hematopoietic cell lineage is also present in *dGCsp*_*ALL*_2 (*IL7R*, *MS4A1*). Furthermore, pathways related to immune cells are also present in *dGCsp*_*ALL*_2. Increase in *STAT3* activity in hematopoietic progenitor cells encourages the production of immature myeloid cells, and increases the amount of plasmacytoid dendritic cells. The amount of immature dendritic cell is also increased. Both immature dendritic cells and plasmacytoid dendritic cells encourage and accumulate regulatory T cells in the tumor microenvironment. *STAT3* activity prevents immature dendritic cells from maturing. However, mature dendritic cells are able to stimulate CD8^+^ T cell's and natural killer cell's anti-tumor effects. *IL7R* and *MS4A1* belong to the lymphoid stem cell branch, and the lymphoid stem cell branch is responsible for the maturing of T and B cell, as seen from the hematopoietic cell lineage in KEGG [33]. From the primary immunodeficiency pathway in KEGG, *LCK* can affect the maturing of T cell, and *BTK* can affect the maturing of B cell. Although *IL7R* and *MS4A1* are involved in the lymphoid stem cell branch, and not the myeloid stem cell, other cross-talk among tumor and immune cells is possible. Note that the plasmacytoid dendritic cells also belong to the lymphoid stem cell branch.

*BTK* also has edges across different pathways. *BTK* can relate to the cross-talk between *STAT3* and *NF-kappaB*, as *BTK* is crucial in the survival of B cell as well as the activation of *NF-kappaB* [44]. *BTK* can also relate to the cross-talk among tumor and immune cells involving hematopoietic progenitor cells since *BTK* plays an important role in the maturation of B cell as mentioned above.

*PTPRCAP*, protein tyrosine phosphatase receptor type C-associated protein, is another vertex that has edges across different pathways. Several protein tyrosine phosphotases, PTPs, have been associated with the regulation of JAKs [45], and the JAK-STAT pathway is important for controlling immune responses [45]. Furthermore, T-cell protein tyrosine phosphatase is identified to be a crucial regulator in the signaling of immune cells [46]. *PTPRCAP* is particularly associated with CD45, an important controller of B and T lymphocyte activation [47]. In *dGCsp*_*ALL*_2, edges are present between *PTPRCAP* and the chemokine receptors, as well as between *PTPRCAP* and the Jak-STAT signaling pathway.

The example from *dGCsp*_*ALL*_2 highlights different cross-talk between pathways or among tumor and immune cells. There can be other cross-talk and interpretations to *dGCsp*_*ALL*_2, yet this proof-of-concept demonstrates that the differential graphlet community approach provides insights to the underlying mechanism and potential treatments for NSCLC. Importantly, the differential graphlet community approach does not only return gene groups, but the edges between them as well. Systematically comparing network structure enables the identification and characterization of differences between tumor and normal samples, and enables the formalization of functional hypotheses and prioritization of biological experiments.

## 4 Concluding remarks

We have developed a graph-based approach that systematically characterizes network structure differences between any graphs, and used it for identifying lung cancer-specific differences between normal and tumor graphs. We proposed using differential graphlet communities for detecting deregulated subgraphs between any graphs. The differential graphlet community approach reveals gene group and wiring differences between compared graphs—in this paper, between normal lung and lung cancer. Going beyond using connectivity of each gene or each edge to compare the identified deregulated subgraphs, we used shortest path distributions on differential graphlet communities in order to exploit network structure information on identified deregulated subgraphs. Importantly, the differential graphlet community approach enables a gene to participate in more than one deregulated subgraph. The ability for overlapping differential graphlet communities is important because genes can have multiple functions in different context. Interestingly, this approach identified difference in network topology between normal and tumor graphs that provided insights to the underlying molecular mechanism in NSCLC. In particular, across all three NSCLC datasets and all three identified differential graphlet communities, a trend that the shortest path lengths are shorter for tumor graphs than for normal graphs is observed; the median of shortest path lengths in normal is significantly larger compared to tumor graphs (adjusted *p* values ≤ 1.13E –20; one-sided Mann–Whitney test). This suggests that tumor cells can create shortcuts between biological processes that may not be present under normal conditions. We have further validated these results on four independent NSCLC datasets. As a proof-of-concept to demonstrate that the differential graphlet community approach provides insights to the underlying mechanism for NSCLC, we highlighted cross-talk between pathways and among tumor and immune cells that are revealed through the systematic graph-based analysis. Examples of cross-talk that are observed include the cross-talk between the Jak-STAT and *NF-kappaB* pathways or *STAT3* signaling enabling cross-talk among tumor and immune cells, resulting in an immunosuppressive network. The systematic network structure comparison enables the identification of network structure differences between tumor and normal samples. The approach can also be extended to compare results across simulated network perturbations, which can be studied in condition-specific manner, and used for predicting effects of altered signaling cascades. Ultimately, this may lead to systems level analysis of drug mechanism of action, and condition-specific prediction of treatment response in precision medicine.

## Abbreviations

KEGG: Kyoto Encyclopedia of Genes and Genomes
NSCLC: non-small cell lung cancer
PPI: protein–protein interaction

## References

[1] de la Fuente A (2010). From ‘differential expression’ to ‘differential networking’—identification of dysfunctional regulatory networks in diseases. Trends Genet.

[2] Cui X, Churchill GA (2003). Statistical tests for differential expression in cDNA microarray experiments. Genome Biol.

[3] Boutros PC, Lau SK, Pintilie M, Liu N (2009). Prognostic gene signatures for non-small-cell lung cancer. Proc. Natl. Acad. Sci. USA.

[4] Jeong H, Mason SP, Barabási AL, Oltvai ZN (2001). Lethality and centrality in protein networks. Nat. Brief Comm.

[5] Pržulj N, Wigle D, Jurisica I (2004). Functional topology in a network of protein interactions. Bioinformatics.

[6] Jonsson PF, Bates PA (2006). Global topological features of cancer proteins in the human interactome. Bioinformatics.

[7] Ideker T, Ozier O, Schwikowski B, Siegel AF (2002). Discovering regulatory and signalling circuits in molecular interaction networks. Bioinformatics.

[8] Chuang HY, Lee E, Liu YT, Lee D, Ideker T (2007). Network-based classification of breast cancer metastasis. Mol. Syst. Biol.

[9] Fortney K, Kotlyar M, Jurisica I (2010). Inferring the functions of longevity genes with modular subnetwork biomarkers of *Caenorhabditis elegans* aging. Genome Biol.

[10] Choi JK, Yu U, Yoo OJ, Kim S (2005). Differential coexpression analysis using microarray data and its application to human cancer. Bioinformatics.

[11] Fuller TF, Ghazalpour A, Aten JE, Drake TA (2007). Weighted gene coexpression network analysis strategies applied to mouse weight. Mamm. Genome.

[12] Watson M (2006). CoXpress: differential co-expression in gene expression data. BMC Bioinformatics.

[13] Qiu P, Wang ZJ, Liu KJR, Hu ZZ, Wu CH (2007). Dependence network modeling for biomarker identification. Bioinformatics.

[14] Zhang B, Li H, Riggins RB, Zhan M (2009). Differential dependency network analysis to identify condition-specific topological changes in biological networks. Bioinformatics.

[15] Pržulj N (2007). Biological network comparison using graphlet degree distribution. Bioinformatics.

[16] Pržulj N, Milenkovi T, Chen J, Lonardi S (2009). Computational methods for analyzing and modeling biological networks. Biological Data Mining.

[17] Pržulj N, Jurisica I, Wigle D (2006). Graph theory analysis of protein-protein interactions. Knowledge Discovery in Proteomics, volume 8 of Chapman and Hall/CRC Mathematical Biology and Medicine Series.

[18] Pržulj N, Corneil DG, Jurisica I (2004). Modeling interactome: scale-free or geometric?. Bioinformatics.

[19] Wong S, Kotlyar M, Strumpf D, Cercone N (2012). Systematic, comparative network analysis on non-small cell lung cancer [abstract].

[20] Garey MR, Johnson DS (1979). Computers and Intractability—A Guide to the Theory of NP—Completeness.

[21] Omidi S, Schreiber F, Masoudi-Nejad A (2009). MODA: an efficient algorithm for network motif discovery in biological networks. Genes Genet. Syst.

[22] Palla G, Derényi I, Farkas I, Vicsek T (2005). Uncovering the overlapping community structure of complex networks in nature and society. Nature.

[23] Hou J, Aerts J, den Hamer B, van Ijcken W (2010). Gene expression-based classification of non-small cell lung carcinomas and survival prediction. PLoS One.

[24] Su L, Chang C, Wu Y, Chen K (2007). Selection of DDX5 as a novel internal control for Q-RT-PCR from microarray data using a block bootstrap re-sampling scheme. BMC Genomics.

[25] Landi MT, Dracheva T, Rotunno M, Figueroa JD (2008). Gene expression signature of cigarette smoking and its role in lung adenocarcinoma development and survival. PLoS One.

[26] Edgar R, Domrachev M, Lash AE (2002). Gene expression omnibus: NCBI gene expression and hybridization array data repository. Nucleic Acids Res.

[27] Lu TP, Tsai MH, Lee JM, Hsu C (2010). Identification of a novel biomarker, SEMA5A, for non- small cell lung carcinoma in nonsmoking women. Cancer Epidemiol. Biomarkers Prev.

[28] Sanchez-Palencia A, Gomez-Morales M, Gomez-Capilla JA, Pedraza V (2011). Gene expression profiling reveals novel biomarkers in nonsmall cell lung cancer. Int. J. Cancer.

[29] Okayama H, Kohno T, Ishii Y, Shimada Y (2012). Identification of genes upregulated in ALK-positive and EGFR/KRAS/ALK-negative lung adenocarcinomas. Cancer Res.

[30] Girard L, Minna JD, Gerald WL, Saintigny P, Zhang L (2011). MSKCC—a primary lung cancer specimens. Gene Express. Omnibus GSE31547.

[31] Brown KR, Otasek D, Ali M, McGuffin MJ (2009). NAViGaTOR: network analysis, visualization and graphing Toronto. Bioinformatics.

[32] Romero P, Wagg J, Green ML, Kaiser D (2004). Computational prediction of human metabolic pathways from the complete human genome. Genome Biol.

[33] Kanehisa M, Goto S (2000). KEGG: kyoto encyclopedia of genes and genomes. Nucleic Acids Res.

[34] Schaefer CF, Anthony K, Krupa S, Buchoff J (2009). PID: the pathway interaction database. Nucleic Acids Res.

[35] Matthews L, Gopinath G, Gillespie M, Caudy M (2009). Reactome knowledgebase of human biological pathways and processes. Nucleic Acids Res.

[36] Bader G, Cerami E, Gross B, Sander C, The Cancer Cell Map http://cancer.cellmap.org/cellmap/home.do.

[37] Cerami EG, Gross BE, Demir E, Rodchenkov I (2011). Pathway commons, a web resource for biological pathway data. Nucleic Acids Res.

[38] Binns D, Dimmer E, Huntley R, Barrell D (2009). QuickGO: a web-based tool for Gene Ontology searching. Bioinformatics.

[39] Grivennikov S, Karin M (2010). Dangerous liaisons: STAT3 and NF-kappaB collaboration and crosstalk in cancer. Cytokine Growth Factor Rev.

[40] Dauer DJ, Ferraro B, Song L, Yu B (2005). Stat3 regulates genes common to both wound healing and cancer. Oncogene.

[41] Bremnes RM, Al-Shibli K, Donnem T, Sirera R (2011). The role of tumor-infiltrating immune cells and chronic inflammation at the tumor site on cancer development, progression, and prognosis: emphasis on non-small cell lung cancer. J. Thorac. Oncol.

[42] Bonecchi R, Galliera E, Borroni EM, Corsi MM (2009). Chemokines and chemokine receptors: an overview. Front Biosci.

[43] Yu H, Kortylewski M, Pardoll D (2007). Crosstalk between cancer and immune cells: role of STAT3 in the tumour microenvironment. Nat. Rev. Immunol.

[44] Shinners NP, Carlesso G, Castro I, Hoek KL (2007). Bruton's tyrosine kinase mediates NF- kappaB activation and B cell survival by B cell-activating factor receptor of the TNF-R family. J. Immunol.

[45] Shuai K, Liu B (2003). Regulation of JAK-STAT signalling in the immune system. Nat. Rev. Immunol.

[46] Doody KM, Bourdeau A, Tremblay ML (2009). T-cell protein tyrosine phosphatase is a key regulator in immune cell signaling: lessons from the knockout mouse model and implications in human disease. Immunol. Rev.

[47] Geer LY, Marchler-Bauer A, Geer RC, Han L (2010). The NCBI BioSystems database. Nucleic Acids Res.

