# Supplementary material for: Comparative network analysis via differential graphlet communities
Source: Proteomics. 2014 Dec 15;15(2-3):608–17. doi: 10.1002/pmic.201400233 (PMC4309523; doi:10.1002/pmic.201400233)
Supplement: Supplementary file 1 [file pmic0015-0608-sd1.docx]

**Supplementary information: Comparative network analysis via differential graphlet communities**

Serene W. H. Wong1*,* 2, Nick Cercone1 and Igor Jurisica*∗*2*,* 3

1 Department of Computer Science and Engineering, York University, Toronto, Canada

2 Princess Margaret Cancer Centre, TECHNA Institute for the Advancement of Technology for Health, UHN, Toronto, Canada

3 Departments of Computer Science and Medical Biophysics, University of Toronto, Toronto, Canada

Email: Serene W. H. Wong - swong@cse.yorku.ca; Nick Cercone - ncercone@yorku.ca; Igor Jurisica*∗* - juris@ai.utoronto.ca;

*∗* Corresponding author

# Materials and methods

## Construction of co-expression graphs.

While the approach is generic, we evaluated it on three NSCLC gene expression datasets. Three NSCLC gene expression datasets and eighteen prognostic NSCLC signatures are the input to the method. The gene expression datasets are described in Section - Datasets, and the prognostic signature information is provided in Additional file 1. The union of genes from all eighteen prognostic gene signatures is denoted as *PS*. For each gene expression dataset, *i*, genes in *i* are intersected with *PS*.

Two co-expression graphs for each dataset, a normal and a tumor graph, are generated using normal and tumor samples, respectively. The co-expression graphs are generated using the following approach, for both normal and tumor samples:

- calculate pairwise Pearson correlations for all gene pairs;
- rank edges according to their absolute correlation values;
- select gene pairs with the top 1% of the absolute correlation values.

## Datasets

| Authors | GSE # | Title | Description |
| --- | --- | --- | --- |
| J. Hou et al. (PLoS One, 2010) | 19188 | Expression data for early stage NSCLC | 91 patients, 91 tumor and 65 adjacent normal lung tissue samples |
| L. Su et al. (BMC Genomics, 2007) | 7670 | Expression data from lung cancer | Pairwise tumor-normal samples from 27 patients |
| M. T. Landi et al. (PLoS One, 2008) | 10072 | Gene expression signature of cigarette smoking and its role in lung adenocarcinoma development and survival | 107 lung adenocarcinoma and normal lung samples, 58 tumor and 49 non-tumor tissues |

Table 1: 3 non-small cell lung cancer datasets are used [1–3].

| Authors | GSE # | Title | Description |
| --- | --- | --- | --- |
| T. P. Lu et al. (Cancer Epidemiol Biomarkers Prev, 2010)   A. Sanchez-Palencia et al.  (Int J Cancer, 2011)  H. Okayama et al. (Cancer Res, 2012)   L. Girard et al. (GSE, 2011) | 19804      18842    31210    31547 | Genome-wide screening of transcriptional modulation in non-smoking female lung cancer in Taiwan  Gene expression analysis of human lung cancer and control samples  Gene expression data for pathological stage I-II lung adenocarcinomas  MSKCC-A primary lung cancer specimens | 120 samples: 60 normal samples, 60 tumor samples  91 samples: 45 controls, 46 tumor samples    246 samples: 20 normal samples, 226 tumor samples   50 samples: 20 adjacent normal lung controls, 30 tumor samples |

Table 2: 4 other distinct non-small cell lung cancer gene expression datasets [4–7].

## Graph theoretical terms

Let *G*(*V, E*) denote a graph where *V* is the set of vertices, and *E*, *E ⊆ V* x *V*, is the set of edges in *G*. A graph is *complete* (called clique) if there exists an edge between all pairs of vertices. Let *x* and *y* be vertices from *G*. *y* is *adjacent* to *x* if there is an edge between *x* and *y*, and *y* is a *neighbour* of *x*. A *path* in a graph that contains no loop contains vertices that can be ordered such that 2 vertices are adjacent if and only if they are consecutive in the ordering. A *subgraph H* of *G* is a graph such that *V*(*H*) *⊆ V*(*G*)*, E*(*H*) *⊆ E*(*G*) and *H* has the same assignment of vertices to edges as in *G*. An *induced subgraph*, *H*, is a subgraph such that *E*(*H*) consists of all edges that are connected to *V*(*H*) in *G*.

## Implementation

The shortest path distribution analysis and the diﬀerential graphlet community analysis were written using the igraph package [8] version 0.5.5.2 in R. The diﬀerential graphlet community analysis adapted the implementation of the clique percolation algorithm in the wiki website of igraph [9]. The Mann-Whitney test was performed in R 2.15.0. The enumeration of all 5-node graphlets was executed using Fanmod [10]. Fanmod is a fast tool to detect network motifs, and contained an algorithm, EnumerateSubgraphs (ESU), by Wernicke [11], to enumerate all size-*n* subgraphs. Graph visualization was from NAViGaTOR version 2.3 - Network Analysis, Visualization, & Graphing TORonto [12].

# Results

Hou

POU2AF1

IL16

C1orf38

Su

POU2AF1 LCK


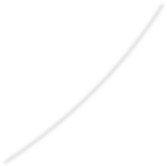

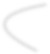

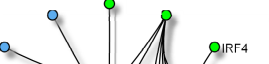

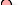

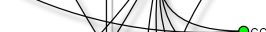

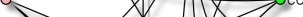

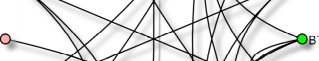

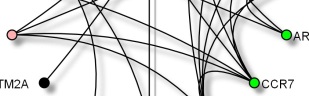

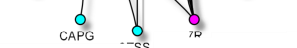


TYR

OBP


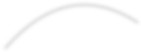

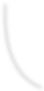

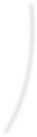

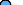

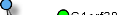

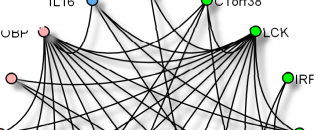

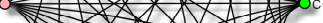

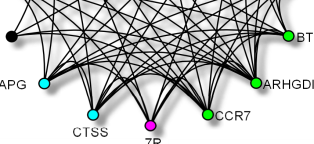


IL16

MS4A1

PTPRCAP

4 TYROBP R2

IRF

CC

R2 MS4A1 TK

CC

B

AR ITM2A

ITM2A

PTPRCAP

HGDIB

C IB

BTK APG ARHGD

I

L7R

L7R

SS

I CT

Landi

POU2AF1

IL16


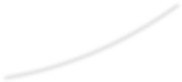

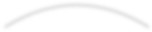

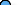

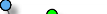

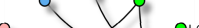

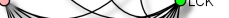

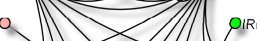

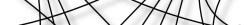

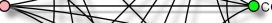

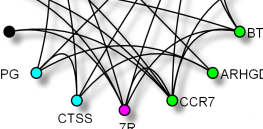


C1orf38

TYROBP

LCK

MS4A1 4

IRF

PTPRCAP R2

CC

BTK PG ARHGD

ITM2A

CA IB

U - Uncharacterized

D - Genome Maintenance

C - Cellular Fate and Organization

P - Translation

B - Transcriptional Control

T - Transcription

M - Other Metabolism

F - Protein Fate

G - Amino Acid Metabolism A - Transport and Sensing

R - Stress and Defence

E - Energy Production

Unmatched

I

CTSS

L7R

Figure 1: *dGCHou*2, *dGCSu*2 and *dGCLandi*2 are shown. Edges connect co-expressed genes. Nodes are sorted and colored based on GO biological function.

Hou Su

TYROBP

ACTA2


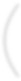

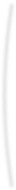

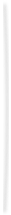

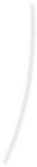

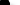

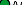

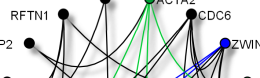

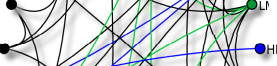

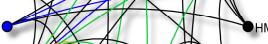

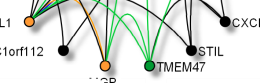


MM T

P2 ZWIN

LM

TYROBP

MEF2

ACTA2

C ZWINT

LM


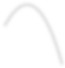

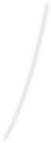

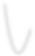

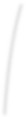

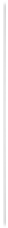

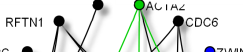

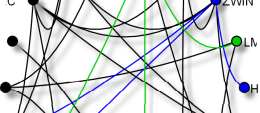

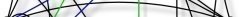

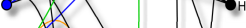

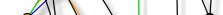

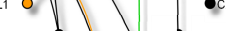

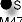

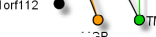


MEF2C

OD1

MMP2

OD1

ARHGDIB RRM2

HN

SPARC

1

MR L12

HM

L1 CXC

ARHGDIB

HN

RRM2

SPARC

L1 CX

1

MMR CL12

H

C1 TIL E

C1orf112

orf112

S

TM

M47

M M

GP

GP

Landi

TYROBP

MMP

ACTA2

2 ZWINT

LM


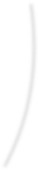

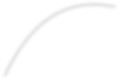

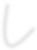

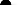

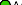

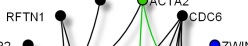

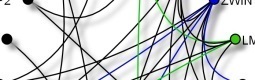

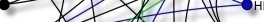

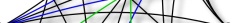

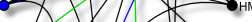

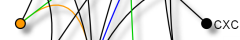

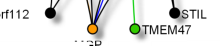


graphlet i

MEF2C ARHGDIB

HN

RRM2

HM

CXC

SPARCL1

OD1

1

MR L12

graphlet j

overlap between graphlets i and j other graphlets

C1

orf112

M

GP

Figure 2: *dGCHou*3, *dGCSu*3 and *dGCLandi*3 are shown. Edges connect co-expressed genes. Differential graphlet communities are formed by graphlets; graphlet *i* is in blue, and graphlet *j* is in green for some *i*, *j* that form *dGC*3. Other graphlets that form *dGC*3 are in black (other graphlets that overlap with graphlets *i*, *j* are not shown).


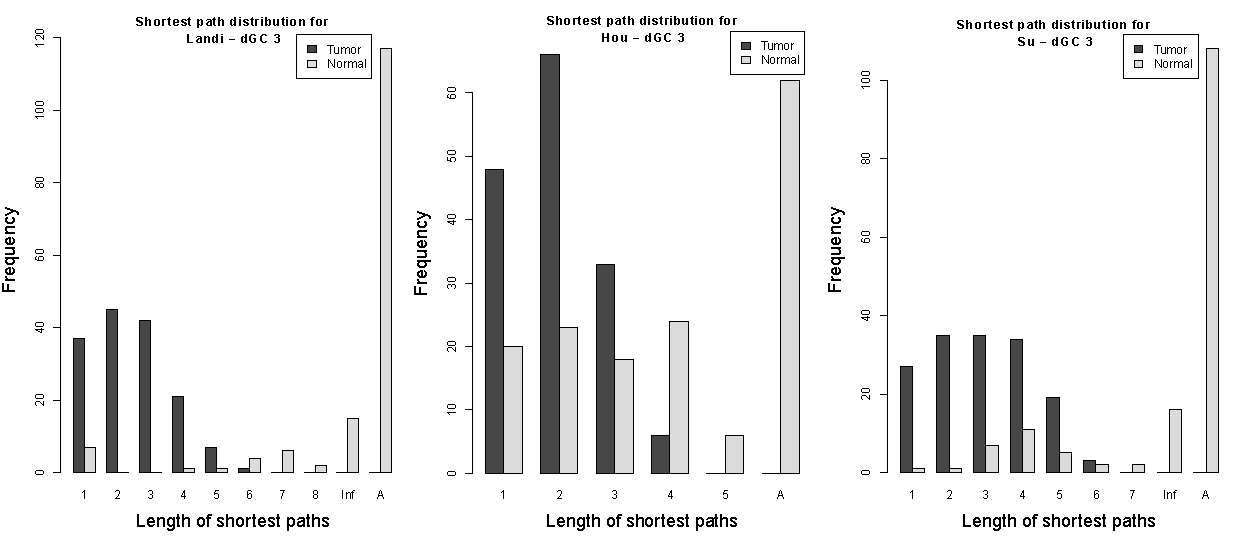


Figure 3: Shortest path distributions for *dGC*3 for Landi, Hou and Su datasets. Inf represents shortest path between unreachable nodes. A is the number of node pairs that have infinity as the distance due to the absence of nodes in the graph.


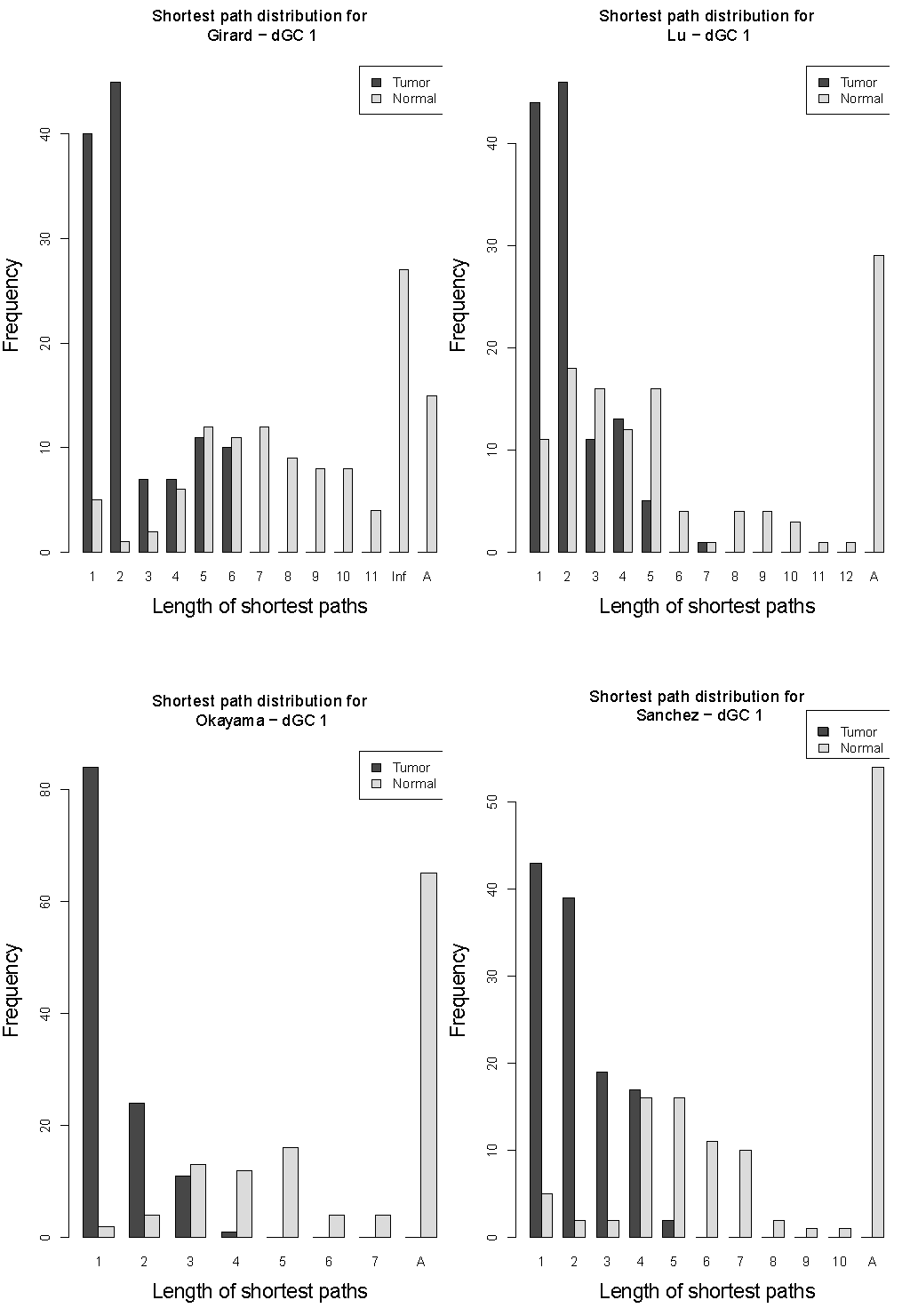


Figure 4: Shortest path distribution for *dGC*1 for Girard and Lu datasets are shown at the top. Shortest path distribution for *dGC*1 for Okayama and Sanchez datasets are shown in the bottom. Inf represents shortest path between unreachable nodes. A is the number of node pairs that have infinity as the distance due to the absence of nodes in the graph.


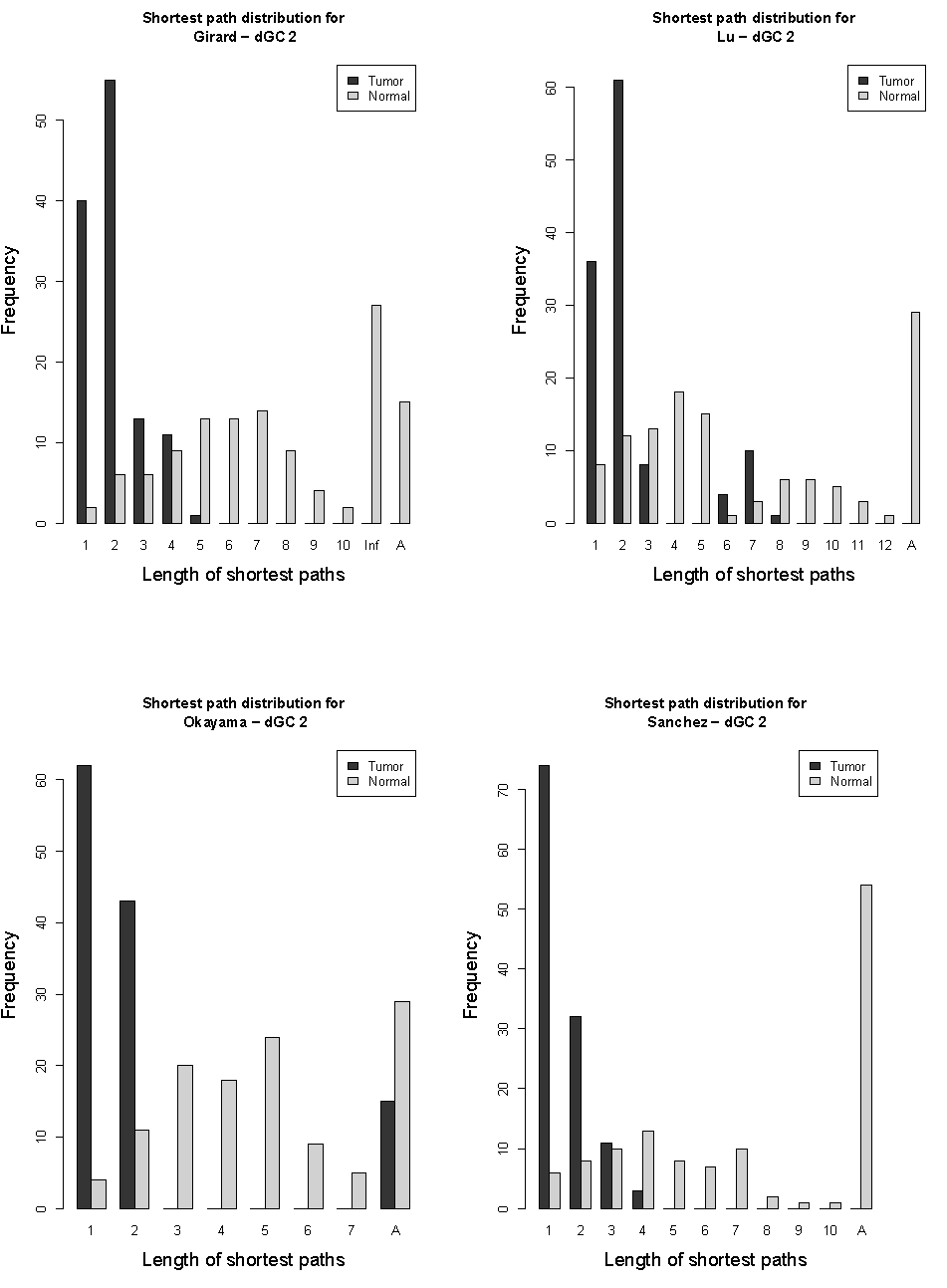


Figure 5: Shortest path distribution for *dGC*2 for Girard and Lu datasets are shown at the top. Shortest path distribution for *dGC*2 for Okayama and Sanchez datasets are shown in the bottom. Inf represents shortest path between unreachable nodes. A is the number of node pairs that have infinity as the distance due to the absence of nodes in the graph.


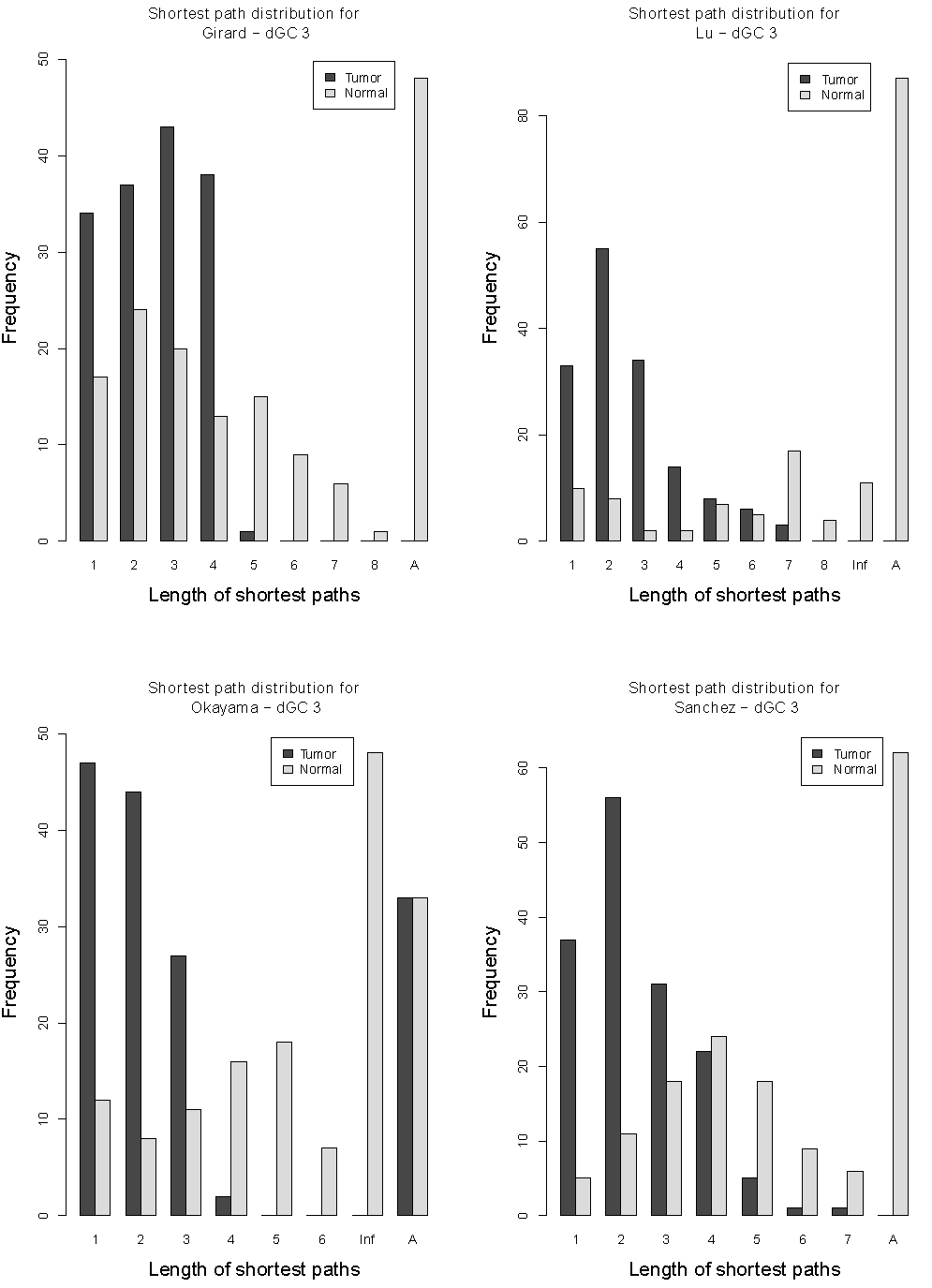


Figure 6: Shortest path distribution for *dGC*3 for Girard and Lu datasets are shown at the top. Shortest path distribution for *dGC*3 for Okayama and Sanchez datasets are shown in the bottom. Inf represents shortest path between unreachable nodes. A is the number of node pairs that have infinity as the distance due to the absence of nodes in the graph.

# Randomization

We have applied the diﬀerential graphlet community approach to three NSCLC datasets, Hou, Su, and Landi, and identified three diﬀerential graphlet communities. We observed a trend that the shortest path lengths are shorter for tumor graphs than for normal graphs between genes that are in diﬀerential graphlet communities. To assess significance of this observation, we ran 1000 randomization tests, and show that the trend that the shortest path lengths are shorter for tumor graphs than for normal graphs between genes that are in diﬀerential graphlet communities is significant (p *<* 0.001, randomization test).

We performed the randomization as follow. Recall that the identified three diﬀerential graphlet communities are referred to as: *dGCHoui, i ∈ {*1*,* 2*,* 3*}* for Hou, *dGCSui, i ∈ {*1*,* 2*,* 3*}* for Su and *dGCLandii, i ∈ {*1*,* 2*,* 3*}* for Landi (as defined in Notations Section of the main text). All shortest paths are computed between all node pairs in *V*(*dGCHoui*), *i ∈ {*1*,* 2*,* 3*}* for *HouN* and for *HouT*. Similarly, for Su and Landi.

For each dataset, we randomly divide the samples into two subsets, denoted as randomA and randomB, 1000 times. Let’s denote *HourandomAj*, *j ∈ {*1*..*1000*}* be the co-expression graph generated using the *jth* randomA samples from Hou, and *HourandomBj*, *j ∈ {*1*..*1000*}* be the co-expression graph generated using the *jth* randomB samples from Hou. Co-expression graphs are constructed as described in the materials and methods section. All shortest paths are computed between all node pairs in *V*(*dGCHoui*), *i ∈ {*1*,* 2*,* 3*}* for *HourandomAj* and for *HourandomBj*, *j ∈ {*1*..*1000*}*. Similarly, for Su and Landi.

For each *j*, for each diﬀerential graphlet community, we compare the shortest path distributions between *HourandomAj* and *HourandomBj* , *SurandomAj* and *SurandomBj* , *LandirandomAj* and *LandirandomBj* using the one-sided Mann-Whitney test, as we did with the real data. In the randomization case, we take the minimum p value out of the 2 p values resulted from the 2 one-sided Mann-Whitney test. The trend that the shortest path lengths are shorter for tumor graphs than for normal graphs between genes that are in diﬀerential graphlet communities is significant (p *<* 0*.*001, randomization test).

# Graph structure

To assess whether the trend – the shortest path lengths are shorter for tumor graphs than for normal graphs between genes that are in diﬀerential graphlet communities – are due to graph structures, we compared the graph structure between the random graphs and the real graphs. We used the *graphlet degree distribution (GDD) agreement* [13], a network similarity measure, to compare the structure between networks. The GDD agreement uses 73 topological properties to compare networks, and one of them is the degree distribution. The GDD agreement is between [0*,* 1], and two networks are similar if the GDD agreement is high. GDD agreement can be calculated using the arithmetic or geometric mean, refer to [13] for detail. We present results for the arithmetic mean in this section.

We computed the GDD agreement for each *HourandomAj*, *j ∈ {*1*..*1000*}* with *HouN*, and for each *HourandomBj*, *j ∈ {*1*..*1000*}* with *HouT*. Similarly, for Su and Landi. For all 6000 random graphs, the average and median of the GDD agreements between the random graphs and the normal or tumor graphs are 0*.*814 and 0*.*812 respectively. The GDD agreement shows that the random graphs are similar to the normal or tumor graphs. Importantly, although the random graphs and the real graphs are similar, the trend that the shortest path lengths are shorter for tumor graphs than for normal graphs between genes that are in diﬀerential graphlet communities is significant (p *<* 0*.*001, randomization test).

# Threshold

In the Materials and methods section, we construct co-expression graphs using the top 1% of the absolute correlation values. Here we investigate if the trend – the shortest path lengths are shorter for tumor graphs than for normal graphs between genes that are in diﬀerential graphlet communities – holds when we use the top 1% *± ε*, *ε ∈ {*0*.*1*,* 0*.*2*}*, of the absolute correlation values.

For the top 0*.*8%, 0*.*9%, 1*.*1%, and 1*.*2%: across all 7 NSCLC datasets and all 3 identified diﬀerential graphlet communities, a trend that the shortest path lengths are shorter for tumor graphs than for normal graphs is observed; the median of shortest path lengths in normal is significantly larger compared to tumor graphs; their adjusted p values are p *≤* 4*.*07*e -* 13, p *≤* 6*.*25*e -* 14, p *≤* 1*.*59*e -* 12 and p *≤* 6*.*78*e -* 10 respectively (one-sided Mann-Whitney test).

# References

1. Hou J, Aerts J, den Hamer B, van Ijcken W, den Bakker M, Riegman P, van der Leest C, van der Spek P, Foekens JA, Hoogsteden HC, Grosveld F, Philipsen S: Gene expression-based classification of non-small cell lung carcinomas and survival prediction. *PLoS One* 2010*, 5*(4).
2. Su L, Chang C, Wu Y, Chen K, Lin C, Liang S, Lin C, Whang-Peng J, SHsu, Chen C, Huang CF: Selection of DDX5 as a novel internal control for Q-RT-PCR from microarray data using a block bootstrap re-sampling scheme. *BMC Genomics* 2007*, 8*(140).
3. Landi MT, Dracheva T, Rotunno M, Figueroa JD, Liu H, Dasgupta A, Mann FE, Fukuoka J, Hames M, Bergen AW, Murphy SE, Yang P, Pesatori AC, Consonni D, Bertazzi PA, Wacholder S, Shih JH, Caporaso NE, Jen J: Gene expression signature of cigarette smoking and its role in lung adenocarcinoma development and survival. *PLoS One* 2008*, 3*(2).
4. Lu TP, Tsai MH, Lee JM, Hsu C, Chen PC, Lin CW, Shih JY, Yang PC, Hsiao CK, Lai LC, Chuang EY: Identification of a novel biomarker, SEMA5A, for non-small cell lung carcinoma in nonsmoking women. *Cancer Epidemiol Biomarkers Prev* 2010*, 19*(10):2590–7.
5. Sanchez-Palencia A, Gomez-Morales M, Gomez-Capilla JA, Pedraza V, Boyero L, Rosell R, Fárez-Vidal ME: Gene expression profiling reveals novel biomarkers in nonsmall cell lung cancer. *Int J Cancer* 2011*, 129*(2):355–64.
6. Okayama H, Kohno T, Ishii Y, Shimada Y, Shiraishi K, Iwakawa R, Furuta K, Tsuta K, Shibata T, Yamamoto S, Watanabe S, Sakamoto H, Kumamoto K, Takenoshita S, Gotoh N, Mizuno H, Sarai A, Kawano S, Yamaguchi R, Miyano S, Yokota J: Identification of genes upregulated in ALK-positive and EGFR/KRAS/ALK- negative lung adenocarcinomas. *Cancer Res* 2012*, 72*:100–11.
7. Girard L, Minna JD, Gerald WL, Saintigny P, Zhang L: MSKCC-A Primary Lung Cancer Specimens. Gene Expression Omnibus GSE31547 2011.
8. Csardi G, Nepusz T: The igraph software package for complex network research. *InterJournal, Complex Systems* 2006, *1695*.
9. Community Detection In R 2012. [[http://igraph.wikidot.com/community-detection-in-r].](http://igraph.wikidot.com/community-detection-in-r)
10. Wernicke S, Rasche F: FANMOD: a tool for fast network motif detection. *Bioinformatics* 2006, *22*(9):1152–1153.
11. Wernicke S: Eﬃcient Detection of Network Motifs. *IEEE/ACM transactions on computational biology and bioinformatics* 2006, *3*(4):347–359.
12. Brown KR, Otasek D, Ali M, McGuﬃn MJ, Xie W, Devani B, van Toch IL, Jurisica I: NAViGaTOR: Network Analysis, Visualization and Graphing Toronto. *Bioinformatics* 2009, *25*(24):3327–3329.
13. Pržulj N: Biological network comparison using graphlet degree distribution. *Bioinformatics* 2007, *23*(2):e177–e183
